# The complete mitogenome of giant freshwater prawn (*Macrobrachium rosenbergii*) from two different selective breeding populations in China

**DOI:** 10.1080/23802359.2021.1938720

**Published:** 2021-06-14

**Authors:** Hongping Li, Minmin Yang, Guozhu Chen, Yunming Wu, Yumei Xiang, Huo Zhu, Kemin Ma, Salifu Ibrahim, Guoliang Yang, Qiongying Tang

**Affiliations:** a College of Life Science, Huzhou University, Huzhou, China; b Jiangsu Shufeng Prawn Breeding Co., LTD, Gaoyou, China

**Keywords:** *Macrobrachium rosenbergii*, mitogenome, selective breeding population, phylogenetic analysis

## Abstract

The giant freshwater prawn (GFP), *Macrobrachium rosenbergii*, is one of the largest freshwater shrimps in the world, being widely cultured because of its high economic value. In this study, complete mitogenomes of two GFP individuals from different selective breeding populations, ‘South Taihu No.2’ (ST) and ‘Shufeng’ (SF), were newly sequenced, compared with each other, and with those of other published *Macrobrachium* species. The total length is 15,767 bp (ST) and 15,766 bp (SF), including 13 protein-coding genes, 22 transfer RNA genes, 2 ribosomal RNA genes, and one control region. The phylogenetic analyses based on whole mitogenome sequences suggest that ‘Shufeng’ has a slightly distant relationship with ‘South Taihu No.2’, with a pairwise genetic distance of 0.011. This study can provide a genetic background for the GFP selective breeding, and add significantly to the knowledgebase regarding crustacean biology and aquaculture as well.

The giant freshwater prawn (GFP), *Macrobrachium rosenbergii* (De Man, 1879), belonging to the family Palaemonidae in the order Decapoda, is widely distributed in the fresh and brackish waters in tropical and subtropical areas of the Indian and Pacific Oceans. It is one of the largest freshwater shrimps in the world, known as the ‘King of Freshwater Shrimp’. The GFP was firstly introduced to China in 1976 and rapidly became an important cultured species because of its high economic value (Yang et al. 2012). For the past 10 years, China has been the top producer of GFP, grossing about 133,000 tons annually which accounts to 50–60% of global production, making China’s GFP culture a principal industry of rural revitalization. This great achievement is attributed to the GFP selective breeding program in China launched in 2006, which yielded one new strain ‘South Taihu No.2’ in 2009. In recent years, another new strain ‘Shufeng’ was developed, and it shows a faster growth and higher survival rate than ‘South Taihu No.2’ (Chen et al. 2013; Sui et al. 2019).

In this study, the complete mitogenomes of two GFP individuals from different selective breeding populations, ‘South Taihu No.2’ (ST) and ‘Shufeng’ (SF), were sequenced by Illumina HiSeq × Ten (GenBank accession number: MW602629 and MW602628). Samples were collected from two different aquatic farms in Sanduo town of Gaoyou city (32°82′N, 119°67′E), Jiangsu province, and preserved in 95% ethanol. Specimens were deposited at Huzhou University (www.zjhu.edu.cn, Qiongying Tang, tangqy@zjhu.edu.cn) under the voucher number HZST201808010 and HZSF201808872). Genomic DNA was extracted from muscles following Tang et al (2008). NOVOPlasty 4.0 (Dierckxsens et al. 2017) and MITOS (http://mitos2.bioinf.uni-leipzig.de/index.py) were used for assembling and annotating the sequenced mitogenome, respectively. Protein-coding genes (PCGs) and rRNAs were also annotated by comparing with the published *Macrobrachium* mitogenomes.

The results show that the newly sequenced mitogenome is 15,767 bp (ST) and 15,766 bp (SF) in total length, slightly shorter than that from Indonesia (15,772 bp, GenBank Accession No: AY659990, herein named as IN) (Miller et al. 2005) and similar with that from China (15,766 bp, KY865098, CN) (Li et al. 2019). Consistent with other crustaceans or fishes (Miller et al. 2005; Shen et al. 2009; Ma et al. 2011; Li et al. 2019; Chen and Tang 2020), the GFP mitogenome is composed of 13 PCGs (*cyt b, ATP6, ATP8, COI-III, ND1-6, ND4L*), 22 tRNA genes, 2 rRNA genes (12S and 16S rRNA), and one control region (D-Loop). Eight tRNAs, *ND1*, *ND5*, *ND4*, *ND4L* and two rRNA genes are encoded on the H-strand, and the other 24 genes are encoded on the L-strand. 9 out of 13 PCGs start with the regular initiation codon ATG but *COI* gene with ACG, *ATP8* and *ND6* with ATC, *ND2* with ATT, which is consistent with other crustaceans, such as *Exopalaemon carinicauda* (Shen et al. 2009) and *Macrobrachium nipponense* (Ma et al. 2011). The initiation codon ATN is typical for metazoan mitogenomes (Wolstenholme 1992). For stop codons, eight PCGs end with TAA or TAG, others using the incomplete stop codon (TA or T). Truncated stop codons TA or T are very common in animal, which are presumably completed as TAA by post-transcriptional polyadenylation (Boore 1999).

The phylogenetic trees of the genus *Macrobrachium* were constructed based on the two newly sequenced and other 16 published whole mitogenomes in the family Palaemonidae. Multiple alignments of sequences were performed using Clustal W2, and the evolutionary model GTR + I + G was selected for phylogenetic analyses. Phylogenetic trees were reconstructed using three methods including neighbor-joining (NJ) in MEGA X.10.2.4, maximum likelihood (ML) in RAxML v. 8.2.12, and Bayesian inference (BI) in MrBayes v. 3.1.2. Three methods yielded nearly identical topologies (Figure 1). All the analyzed *Macrobrachium* species forms a monophyly with high support values (100 or 1.00). All *M. rosenbergii* samples clustered together, and the individuals ST and CN formed a sister group with 0.002 of pairwise genetic distance, indicating that ST has a closer relationship with CN than SF. Probably, both ST and CN came from the same selective breeding population ‘South Taihu No.2’. The genetic distance between ST and SF is 0.011, portraying a slightly distant relationship between the two populations. The present study can provide a genetic background for the GFP selective breeding, and add significantly to the knowledgebase regarding crustacean biology and aquaculture as well.

**Figure 1.**
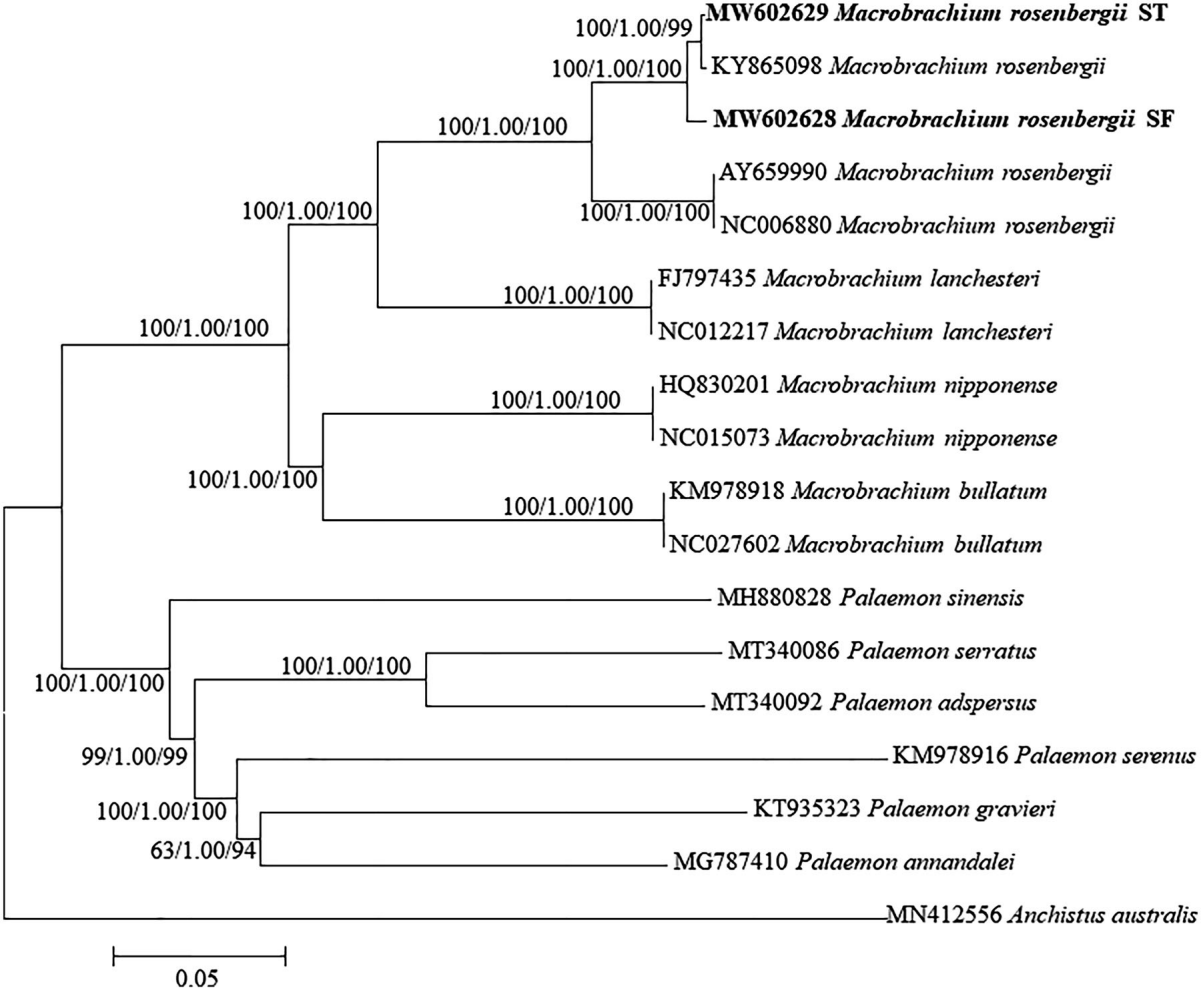
Phylogenetic tree of the genus *Macrobrachium* reconstructed using neighbor joining (NJ), Bayesian method (BI), and maximum-likelihood (ML) based on whole mitogenome sequences. Values at the nodes correspond to the support values for NJ/BI/ML methods. Bold type indicates the newly sequenced mitogenome.

## Data Availability

The genome sequence data that support the findings of this study are openly available in GenBank of NCBI at https://www.ncbi.nlm.nih.gov/, under the accession no. MW602628-MW602629.
